# Myo1e Impairment Results in Actin Reorganization, Podocyte Dysfunction, and Proteinuria in Zebrafish and Cultured Podocytes

**DOI:** 10.1371/journal.pone.0072750

**Published:** 2013-08-19

**Authors:** Jianhua Mao, Dayan Wang, Parikka Mataleena, Bing He, Dadi Niu, Kan Katayama, Xiangjun Xu, Juha RM Ojala, Wenjing Wang, Qiang Shu, Lizhong Du, Aimin Liu, Timo Pikkarainen, Jaakko Patrakka, Karl Tryggvason

**Affiliations:** 1 Department of Nephrology, The Children’s Hospital of Zhejiang University School of Medicine, Hangzhou, P. R. China; 2 Division of Matrix Biology, Department of Medical Biochemistry and Biophysics, Karolinska Institute, Stockholm, Sweden; Fondazione IRCCS Ospedale Maggiore Policlinico & Fondazione D’Amico per la Ricerca sulle Malattie Renali, Italy

## Abstract

**Background:**

Podocytes serve as an important constituent of the glomerular filtration barrier. Recently, we and others identified Myo1e as a key molecular component of the podocyte cytoskeleton.

**Results:**

Myo1e mRNA and protein was expressed in human and mouse kidney sections as determined by Northern blot and reverse transcriptase PCR, and its expression was more evident in podocytes by immunofluorescence. By specific knock-down of *MYO1E* in zebrafish, the injected larvae exhibited pericardial edema and pronephric cysts, consistent with the appearance of protein in condensed incubation supernate. Furthermore, specific inhibition of Myo1e expression in a conditionally immortalized podocyte cell line induced morphological changes, actin cytoskeleton rearrangement, and dysfunction in cell proliferation, migration, endocytosis, and adhesion with the glomerular basement membrane.

**Conclusions:**

Our results revealed that Myo1e is a key component contributing to the functional integrity of podocytes. Its impairment may cause actin cytoskeleton re-organization, alteration of cell shape, and membrane transport, and podocyte drop-out from the glomerular basement membrane, which might eventually lead to an impaired glomerular filtration barrier and proteinuria.

## Background

The primary barrier for ultrafiltration of plasma in renal glomeruli is composed of three layers: a fenestrated endothelium, a 300-350 nm thick glomerular basement membrane (GBM), and slit pores, i.e., diaphragms located between the foot processes of the epithelial podocytes. The glomerular slit diaphragm plays a crucial role in preventing the loss of circulating macromolecules into the urine. This barrier is a highly sophisticated size-selective molecular sieve whose molecular mechanisms of function are still largely unclarified [1–3].

Class I myosins are single-headed, actin binding, mechanochemical “motor” proteins with heavy chains in the molecular mass range of 110-130 kDa and they do not form filaments [4,5]. Each myosin I heavy chain is associated with one to six light chains that bind to specific motifs known as IQ domains. Mice and humans have a total of eight class I myosin heavy-chain genes, six of which encode short-tailed forms (Myo1a, b, c, d, g, and h) and two of which encode long-tailed forms (Myo1e and f) [6]. Myo1e is the only long-tailed class I myosin that is ubiquitously expressed in mammalian cells [7,8].

Recently, Myo1e was identified to play an important role in podocyte and kidney function. Complete knockout of Myo1e in mice [9] and *MYO1E* mutations (A159P and Y695X) in humans are associated with nephrotic syndrome and focal segmental glomerulosclerosis [10,11]. Moreover, Chase et al. [12] reported that conditional knockout of Myo1e in podocytes disrupts the integrity of the glomerular filtration barrier, and results in proteinuria, podocyte foot process effacement, and glomerulonephritis.

We recently identified more than 300 glomerulus-upregulated transcripts through large scale sequencing and microarray profiling of the glomerular transcriptome. Among these transcripts, four class I myosin transcripts, Myo1b, Myo1c, Myo1d, and Myo1e, were identified in microarray analyses and their expression profiles were characterized in detail. Ultimately, Myo1e was verified to be a key component contributing to the functional integrity of podocytes in zebrafish, mouse cultured podocytes, and human kidney.

## Materials and Methods

### Reverse Transcription PCR

The expression of *MYO1E* transcripts was studied using reverse transcription PCR (RT-PCR). Primer sequences and sizes of expected PCR products were listed in Table 1.

**Table 1:** Sequences for all primer sets, morpholino, and shRNA.

	Sequence	
RT-PCR	Upstream: 5’-GAGCCATGAGCACTTCAACA-3’	Product size: 414 bp
	Downstream: 5’-ACACGCCGATAAGCATAACC-3’	
Morpholino	5´-CTGAGCCTGAGAAACACAGAGAGAT-´3	*MYO1E-1*
	5´-AGTGATATTTCTCCTTGCTCCCCAT-´3	*MYO1E-2*
	5´-CCTCTTACCTCAGTTACAATTTATA-´3	Control
shRNA	Hairpin sequence: GATCCGAACCCGCCTCATATCTATTTCA	sc-44614-SHA
	AGAGAATAGATATGAGGCGGGTTCTTTTT	
	Corresponding siRNA sequences	
	Sense: GAACCCGCCUCAUAUCUAUtt	
	Antisense: AUAGAUAUGAGGCGGGUUCtt	
	Hairpin sequence: GATCCGCATGGACTACTATTACTATTCAAGAG	sc-44614-SHB
	ATAGTAATAGTAGTCCATGCTTTTT	
	Corresponding siRNA sequences	
	Sense: GCAUGGACUACUAUUACUAtt	
	Antisense: UAGUAAUAGUAGUCCAUGCtt	
	Hairpin sequence: GATCCCCATGAATGTGATTGGAATTTCAAGAG	sc-44614-SHC
	AATTCCAATCACATTCATGGTTTTT	
	Corresponding siRNA sequences:	
	Sense: CCAUGAAUGUGAUUGGAAUtt	
	Antisense: AUUCCAAUCACAUUCAUGGtt	

**Figure 1:**
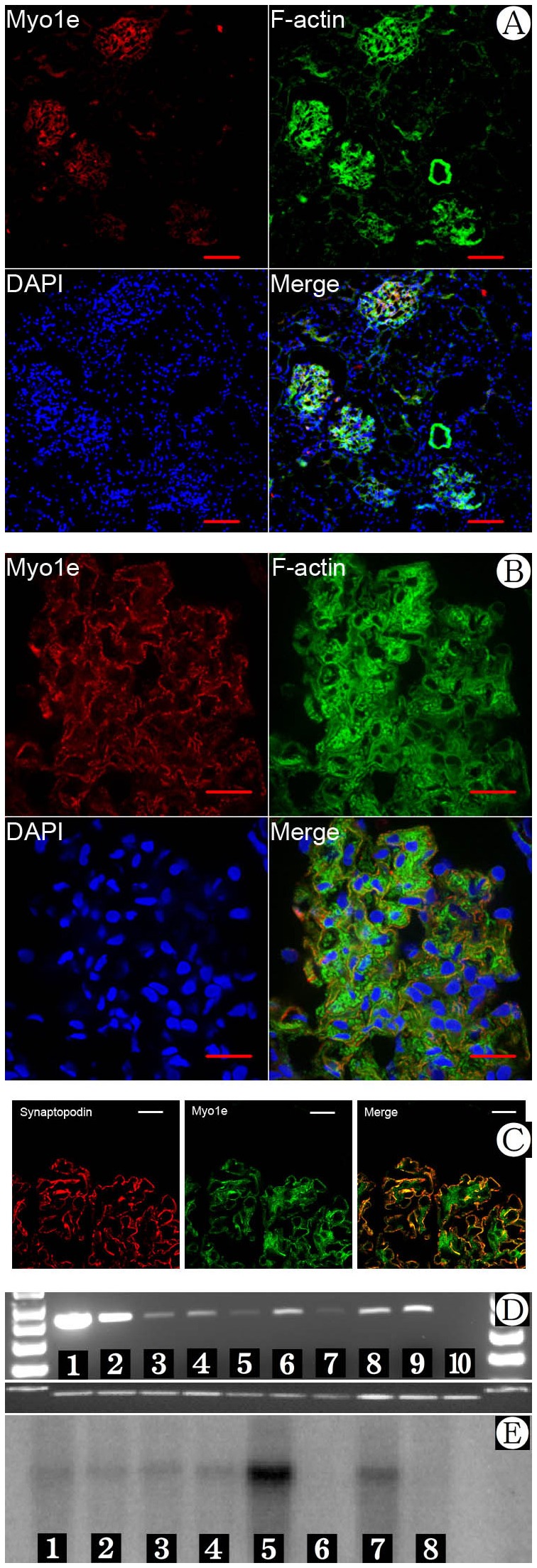
Expression of Myo1e in human and mouse tissues. A) Myo1e expression in normal human kidney. Scale bar: 100μm. B) Myo1e expression in normal human kidney. Scale bar: 20μm. C) Co-expression of synaptopodin and Myo1e in normal human podocytes observed by immunofluorescence and confocal microscope. Scale bar: 10μm. D) mRNA expression of Myo1e in multiple mouse tissues by RT-PCR. 1=purified glomeruli, 2=kidney without glomeruli, 3=lung, 4=brain, 5=testis, 6=spleen, 7=heart, 8=muscle, 9=total kidney, 10=β-actin. Myo1e was expressed abundantly in kidney (both in glomeruli and other tissue within the kidney) E) mRNA expression of Myo1e in multiple mouse tissues by Northern blot. 1=heart, 2=brain, 3=spleen, 4=lung, 5=liver, 6=muscle, 7=total kidney, 8=testis.

As a template for PCR analysis, we used cDNA libraries that were generated from various adult mouse tissues (Figure 1C, band 3^rd^ to 10^th^ with lung, brain, testis, spleen, heart, muscle, total kidney, and β-actin) (Mouse Multiple Tissue cDNA Panel I; Clontech Laboratories, Palo Alto, CA). RT-PCR was also performed on the mRNA isolated from mouse kidney fractions that contained either only glomerular tufts or the kidney excluding glomeruli (Figure 1C band 1^st^ and 2^nd^) in our own laboratory. Glomeruli were isolated from adult ICR outbred mice (Charles River Laboratories, Wilmington, MA) by means of a magnet beads perfusion method [13,14]. RNA from isolated glomeruli and the other part of kidney was extracted by the kit purchased from Invitrogen (TRIzol^®^ kit) and used for cDNA synthesis with the aid of the Sensiscript RT Kit (Qiagen, Westburg BV, Leusden, The Netherlands). RNA samples were treated with RNase-free DNase. The total RNA was used to synthesize cDNA with the Superscript First-Strand synthesis system for RT-PCR. PCR amplification was carried out with TaqDNA polymerase (Invitrogen, Carlsbad, CA), and the amplified fragments were analyzed on 1.5% agarose gel.

### Northern blot

cDNA probes obtained from the amplification of cDNA libraries (see previous RT-PCR paragraph) were used. The probes were ^32^P-labeled using the Rediprime II random primer labeling system (Amersham Pharmacia Biotech, London, UK), and the probes were hybridized to the blots that contained RNA isolated from various mouse organs (Mouse MTN Blot; Clontech Laboratories). The hybridizations were performed according to standard procedures. As a positive loading control, we used a glyceraldehyde-3-phosphate dehydrogenase probe (Clontech Laboratories).

### Immunofluorescence

The methods and primary antibodies used have been previously described [14]. We raised antisera directed against novel glomerular proteins by purifying recombinant proteins with affinity tags and by immunizing New Zealand white (NZW) rabbits with these antigens using standard protocols (SVA, Uppsala, Sweden; KTH, Stockholm, Sweden). Briefly, for the production of Myo1e antigen, we generated mouse recombinant proteins. Myo1e residues 50 to 262 were cloned into the pET-28a(+) expression vector (Novagen, Madison, WI). The his-tagged Myo1e recombinant proteins were solubilized from inclusion bodies in 8 M urea. Then, Myo1e antigen was purified using sequential S-Sepharose ion exchange and Sephadex S-200 gel filtration columns (Amersham Biosciences).

For double-labeling experiments, we used anti-mouse synaptopodin (Progen, Heidelberg, Germany), and anti-human nephrin 50A9 antibodies [14,15]. Secondary antibodies were purchased from Jackson ImmunoResearch Laboratories (West Grove, PA). Microscopy was performed with a Leica standard fluorescent microscope or a standard Leica confocal laser scanning microscope. For immunofluorescence staining, kidney sample were collected from either adult human cadaver kidneys that were unsuitable for transplantation (obtained from the IV department of Surgery of Helsinki, Finland under the body donation scheme. Meanwhile, the donor or their relatives signed the consent before the use for research purposes) or patients with congenital nephrotic syndrome of the Finish type (from the IV department of Surgery of Helsinki, Finland). Informed written consent was obtained from patients [14].

### Zebrafish lines

Wild-type zebrafish were maintained and raised as described [16]. The wild-type lines were of the AB background. Dechorionated embryos were kept in E3 solution with or without 0.003% PTU (1-Phenyl-2-thiourea, Sigma) to suppress pigmentation and staged according to hpf. Whole embryos were observed using a Nikon SMZ 645 or Leica MZ12 dissecting stereomicroscope, the latter with an attached digital camera (Spot Insight QE, USA) and processed with Adobe Photoshop software (Adobe, Inc.).

### Morpholino antisense oligonucleotides

Two different morpholino antisense oligonucleotides (MOs) were designed against the translational initiation site or splice blocking sequence of zebrafish Myo1e. The MOs were obtained from GENE TOOLS, LLC, Philomath, OR. The following sequences of morpholinos were used: *MYO1E-1* (Exon 3 Splice Blocking) 5´-CTGAGCCTGAGAAACACAGAGAGAT-´3. *MYO1E-2* (Translational Blocking) 5´-AGTGATATTTCTCCTTGCTCCCCAT-´3 and *Control oligo*: 5´-CCTCTTACCTCAGTTACAATTTATA-´3 (Table 1).

The morpholinos were diluted in injection solution containing 100 mM KCl and 0.1% phenol red (Sigma), and titrated to determine the lowest concentration sufficient to consistently induce abnormal mRNA splicing. Two morpholinos for Myo1e were injected into one-cell stage embryos. The injection volume was approximately 3 nl containing 125 μM of anti-Myo1e MO. For each morpholino, more than 300 individual embryos were injected. We raised morpholino injected embryos, and observed the phenotypes with stereoscopes. Injections were performed using a microinjector PLI-90 (Harvard Apparatus, Cambridge, MA).

### Morphant characterization

Zebrafish embryos were observed under a stereoscope from day 2 to 5 day post fertilization (dpf). The zebrafish nephron is located beneath somite 3-4, medial to the pectoral fins. Normally, they are invisible against a transparent background. However, cysts are clearly visible as bubbles in this region.

### Histology

Embryos were fixed in 4% PFA at 4°C overnight. After washing in PBS dehydrated in gradient ethanol, they were embedded in JB-4 resin (Polysciences Inc.) overnight at room temperature and the sectioned at 3-5 μm. Slides were then stained with hematoxylin and eosin.

### Detection of proteinuria in zebrafish embryo

A new method was developed to assess the amount of proteinuria in zebrafish embryos. For each measurement, 100 wild-type, control, nephrin MO, and *Myo1e* bryos (AB) were collected at 96 hpf and kept in tanks with 5 ml of fresh E3 water for 24 hours at 28.5°C after changing E3 water twice. At 120 hpf, 4 ml of E3 water were harvested after ensuring that all embryos were alive. One ml of 100% TCA solution was mixed gently with the tank water and kept at 4°C for 1 hours. The samples were then centrifuged at 13,000 rpm at 4°C for 5 minutes, and the supernatant was removed. The pellets were washed with cold acetone and centrifuged at 13,000 rpm at 4°C for 5 minutes twice. After drying the pellets, 15 μl of 2× NuPAGE LDS sample buffer (Invitrogen) was added and incubated at 70 °C for 10 minutes. For SDS-PAGE, the samples were applied to NuPAGE 4 to 12% Bis-Tris Gels (Invitrogen). Then the gels were stained with Page-Blue Protein Staining Solution (Fermentas International Inc.) and examined for the presence of protein by Mass Spectrum [17].

### Rescue experiment

To verify whether or not the phenotype observed in zebrafish embryos was due to the down-expression of *Myo1e* specifically, the co-injection of target morpholino and the 0.02µg/µl mouse *Myo1e* mRNA (by GenePharma Co., Ltd, Shanghai, China) was performed simultaneously. The method for the generation and characterization of the podocin-GFP transgenic zebrafish line was conducted by He et al and published recently [18].

### Podocyte culture

The conditionally immortalized mouse podocyte cell line was kindly provided by Dr. Peter Mundel (Mount Sinai School of Medicine, New York, NY). To propagate podocytes, cells were cultured at 33°C in RPMI 1640 medium supplemented with 10% fetal bovine serum and 10 U/ml mouse recombinant interferon-γ (R&D Systems, Minneapolis, MN) to enhance the expression of a thermosensitive T antigen. To induce differentiation, podocytes were grown under nonpermissive conditions at 37°C in the absence of interferon-γ for 14 days.

### Transfection with shRNA

Myo1e shRNA plasmid (m) is a pool of three different shRNA plasmids: sc-44614-SHA, sc-44614-SHB and sc-44614-SHC. All sequences were listed in Table 1 and all sequences are provided in 5′ → 3′ orientation (Santa Cruz Biotechnology, Inc.). The vector used was the sc-44614-SH plasmid. It is said that the company has never provided the vector sequence to any customer.

The first shRNA treatment was applied when the cells were 50-70% confluent (as determined with a voltmeter). Next, 1.5×10^6^ cells were washed twice with 2 ml of shRNA transfection medium. For each transfection, 0.8 ml of shRNA plasmid transfection medium was added to each well. Then, 200 µl of shRNA plasmid DNA/shRNA plasmid transfection reagent complex (Santa Cruz Biotechnology, Inc.) was added to each well and the plates were incubated for 7 h at 37°C in a CO_2_ incubator. Following incubation, 1 ml of normal growth medium containing two times the normal serum and antibiotic concentration (2× normal growth medium) was added and the cells were incubated for an additional 24 h. For transient transfection, the media was aspirated and replaced with fresh 1× normal growth medium. The cells were assayed using the appropriate protocol 48 h after the addition of fresh medium.

### MTT cell proliferation

The [3-(4,5-dimethythiazol-2-yl) -2,5-diphenyl tetrazolium bromide] MTT Cell Proliferation Kit I (Roche Diagnostics, IN, USA) was used to investigate the proliferation ability of podocytes. Different groups of cells were seeded into 96-well culture plates and incubated at 37.0°C with RPMI 1640 for 144 h. At every 24h, the 20 µl MTT reagent was added to each well in different tissue culture plates and incubated for additional 4 hr in humidified atmosphere (37.0°C, 5% CO_2_). The excess culture solution was removed, and 150 µl of the solubilization solution (DMSO) was added to each well and measured at 570 nm. Results were expressed as percentage of controls.

### Transwell migration assay

24-well tissue culture plates with inserts containing an 8-μm pore-size polycarbonate membrane (Falcon) were used. MPC5s at first passage were quiesced for 72 h, trypsinized, and resuspended in RPMI 1640 supplemented with 0.25% bovine serum albumin (migration medium). MPC5s and culture medium were subsequently loaded into the upper wells (2×10^4^ cells in 100 μl) and incubated for 7 h at 37°C. Serum containing medium as a chemoattractant were placed in the well below. The cell suspension was aspirated, and the membranes were fixed with paraformaldehyde. MPC5s were removed from the upper surface of the membranes, rinsed in water and stained for 10 min in crystal violet. The membranes were then detached from the inserts and mounted for microscopic examination. Cell nuclei on the underside of each membrane were counted in 12 random high power fields (×200) under a light microscope. MPC5 migration in the experimental groups was expressed as mean number of migrated cells per high power field.

### Endocytosis

Cells were incubated with 50 µg/ml FITC-transferrin (Molecular Probes) in PBS for 15 min at 37°C. Coverslips were washed twice with ice-cold PBS. To remove cell surface transferrin, cells were incubated for 2 min at 37°C in a pH 4.6 citrate buffer, and re-equilibrated with two additional ice-cold PBS washes. The coverslips were fixed in a 4% formaldehyde solution at room temperature for 15 min. Excess formaldehyde was removed with 3-5 min washes in PBS. Observations were made under a fluorescence microscope.

### Adhesion assays

Six-well plates were coated with collagen I (10 μg/ml), with 1% bovine serum albumin as the control. Podocytes were harvested with trypsin/EDTA and resuspended in serum-free medium. Cells were allowed to attach at 37°C for 1 h. Unbound cells were removed by washing twice with PBS. Attached cells were fixed in 4% paraformaldehyde and counted. Two individuals recorded cell attachment for these experiments. Cell counts were obtained by averaging the cell numbers from five wells.

### Detachment assay

Wild type and *MYO1E* knock-down podocytes were evaluated after PAN-induced detachment. All cells were cultured on six-well cell culture dishes under nonpermissive conditions for 14 days before analysis. Fields of cells were marked, and cell numbers per field were counted to establish a baseline number. The cells were treated with 50 μg/ml PAN, and cell numbers were counted for each field at 24 h. The average numbers of four fields in three independent sets of experiments were analyzed by student’s *t* test.

To compare the differences among control, negative control and MYO1E knock-down groups in cultured podocytes, each of the following assays was repeated three times as three independent experiments: real-time PCR & Western blot, MTT assay and endocytosis assay. Further, transwell migration assay, adhesion assays and detachment assay were conducted in 6-well cell culture plate, and cell number in each well was counted respectively for further statistical analysis.

### Ethical Considerations

This study was approved by the ethical committees of the Karolinska Institute in Sweden and the ethical committees of Zhejiang University in China.

## Results

### Myo1e expression in glomerular podocytes

Staining of human kidney sections with antibodies against Myo1e revealed that in the kidney, Myo1e was predominantly expressed in the glomeruli (Figure 1A, 1B, 1F). Myo1e partially co-localized with synaptopodin, a podocyte-specific marker, in podocyte foot process (Figure 1C). In addition to foot process staining, Myo1e exhibited prominent mesangial region staining, which was more evident in kidney sections from patients with congenital nephrotic syndrome of the Finnish type (CNF) (Figure 1B). To verify the specificity of the antibody used in present study, Myo1e expression was studied in heart (Figure S1-A), liver (FigureS1-B), spleen (Figure S1-C) and negative control glomeruli (Figure S1-D) by immunofluorescence.

Northern blot and reverse transcription polymerase chain reaction (RT-PCR) assay of mouse organs (liver, testis, lung, spleen, brain, muscle, heart, and kidney) confirmed that Myo1e was significantly expressed in the kidney. Figure 1D demonstrates expression of Myo1e transcripts in various mouse organs by RT-PCR and Northern blot (Figure 1E). In Northern blot analysis, the Myo1e probe hybridized a strong band in liver mRNA, whereas a weaker band was also observed in kidney mRNA. Myo1e mRNA was detected in all organs by RT-PCR, whereas it was present more abundantly in the kidney, especially the glomeruli purified from mouse kidney [14,19,20].

### Myo1e knock-down zebrafish have kidney disease

We designed two morpholinos against *MYO1E* with different sequences in order to test its requirement in glomerular function in zebrafish. Gene specific morpholino were injected respectively into 1-2 cell stage zebrafish embryos and allowed the embryos to develop to 96 hours post-fertilization (hpf). By this time, fenestrated endothelia, GBM, regularly spaced podocyte foot processes, and slit diaphragms were all present indicating that the glomerular filtration barrier is relatively mature [21]. We anticipated that loss of glomerular function would be associated with pericardial edema, based on reported knockdown of the nephrin and podocin genes in zebrafish, and the analysis of zebrafish morphants and mutants [21–23].

**Figure 2:**
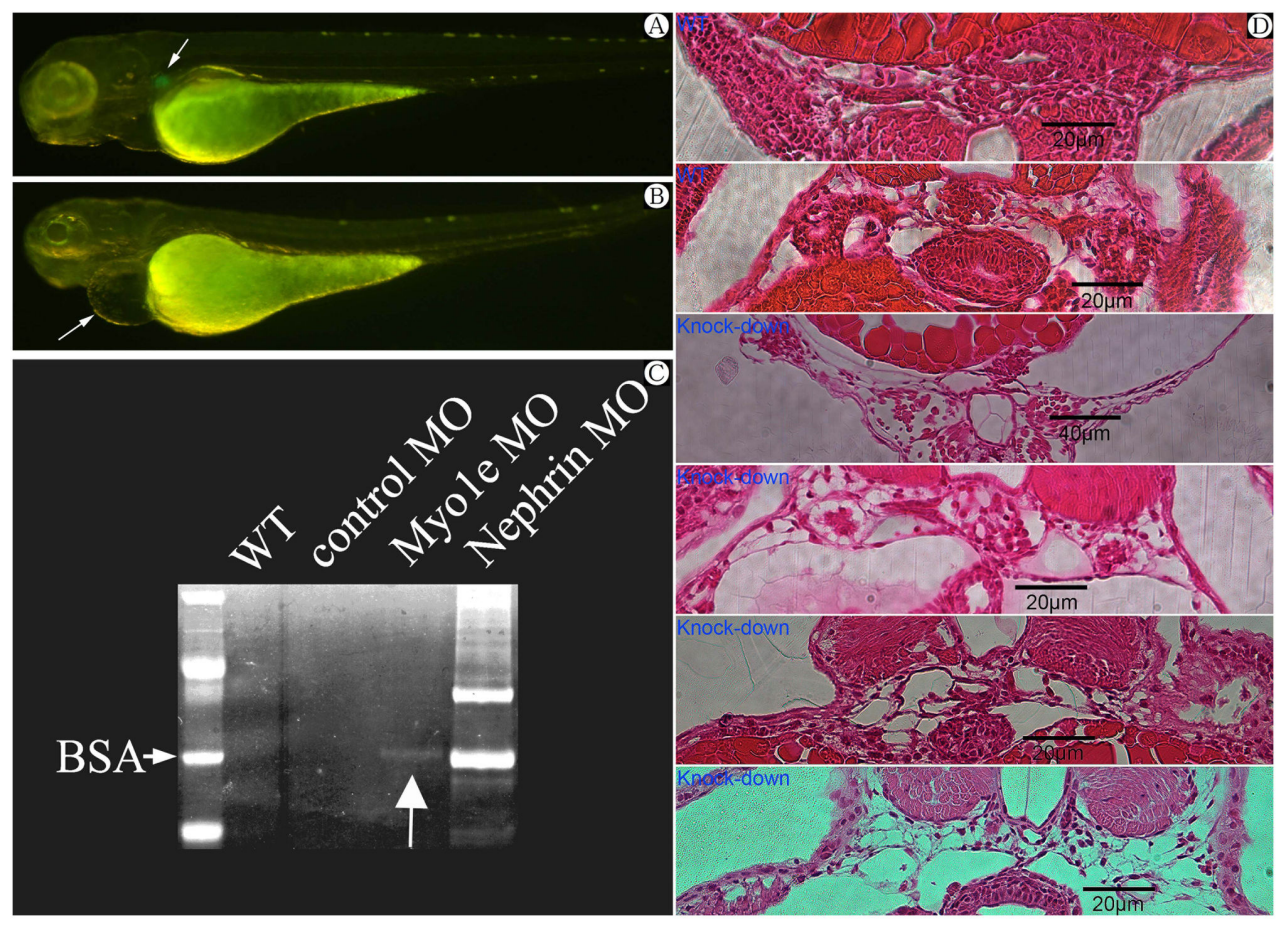
A) Wild type zebrafish at 4 day post fertilization (dpf).Arrow indicating the glomeruli with visible GFP. B) Phenotype of Myo1e morphants at 4 dpf (days post fertilization) with Myo1e specific knock-down morpholino injected. Note the pericardial edema (arrow) and invisible GFP indicating the destruction of glomerular structure. C) Proteinuria (arrow indicating Vitellogenin at 70kD band) in concentrated incubation medium of zebrafish larvae after *MYO1E* knock-down. BSA: bovine serum albumin. D) Histological sections at the level of the glomerulus in zebrafish injected Myo1e MO. The expanded Bowman’s space and destroyed glomeruli observed in *MYO1E* knock-down zebrafish compared with wild type zebrafish.

Finally, the *MYO1E* knock-down resulted in pericardial edema (Figure 2B), an expanded Bowman’s space, and pronephric cyst phenotypes (Figure 2C), consistent with the inability to osmoregulate. The penetrance of these phenotypes varied from 65% to 76% depending on the efficacy of the morpholino and time of injection. A standard control morpholino was injected and served as a baseline for the occurrence of spontaneous pericardial edema. For the larvae injected with the control morpholino, the penetrance of these phenotypes was < 2% to 4%.

To confirm the functional conservation of Myo1e in the maintenance of the podocyte filtration barrier, we assayed barrier function by comparing the protein contained in the supernate of incubation medium between MYO1E knock-down zebrafish larvae, nephrin knock-down zebrafish, and controls. After concentration, the supernate was examined with SDS-PAGE electrophoresis and CBB staining. Compared with the result from the incubation supernate of nephrin knock-down larvae, a weak but evident 70 kD-band, which turned out to be vitellogenin [17] by mass spectrometry (a transport protein highly abundant in the yolk and in blood of zebrafish) could be seen in the condensed incubation supernate of MYO1E knock-down larvae, and vitellogenin was absent in the condensed incubation supernate of the wild type larvae and control morpholino larvae (Figure 2D). The results confirmed the existence of proteinuria of *MYO1E* knock-down zebrafish larvae during development.

Furthermore, with the injection of rescuing Myo1e mRNA, 76.1% (121 in 159) zebrafish presented without pericardial edema, only 23.9% zebrafish presented with pericardial edema and simultaneously GFP loss [18] within glomerular area, compared with 39.2% zebrafish presented with pericardial edema and simultaneously GFP loss after injection of specific morpholinos at same time (χ^2^=31.072, P<0.001).

### Morphological change and dysfunction of *MYO1E* knock-down podocytes in culture Myo1e contributes to podocyte morphology and cytoskeletal integrity

**Figure 3:**
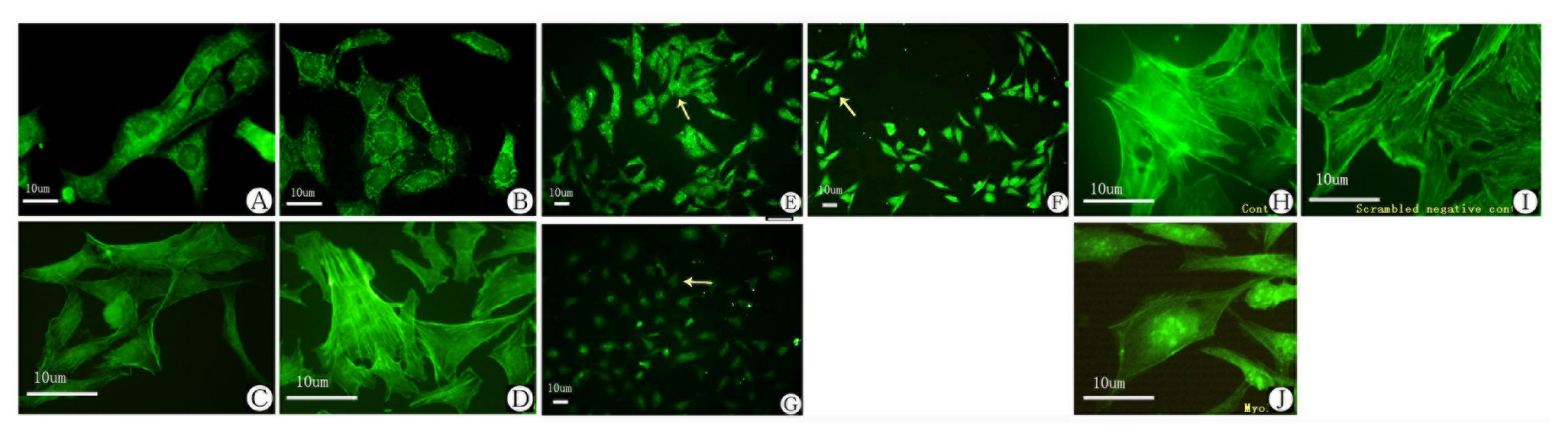
Myo1e and F-actin expression in cultured podocytes. A) Myo1e expression in proliferative MPC5 at 33°C. B) Myo1e expression in differentiated MPC5 at 37°C. C) F-actin expression in proliferative MPC5 at 33°C. D) F-actin expression in differentiated MPC5 at 37°C. E) Myo1e expression in MPC5 (control). F) Myo1e expression in MPC5 (scrambled negative control). G) Myo1e expression in MPC5 after shRNA knock-down. Arrow: Myo1e expression was down-regulated in G than that in E & F.H) F-actin expression in controlled podocytes. I) F-actin expression in scrambled negative control podocytes. J) Change of F-actin organization in *MYO1E* knock-down podocytes.

**Figure 4:**
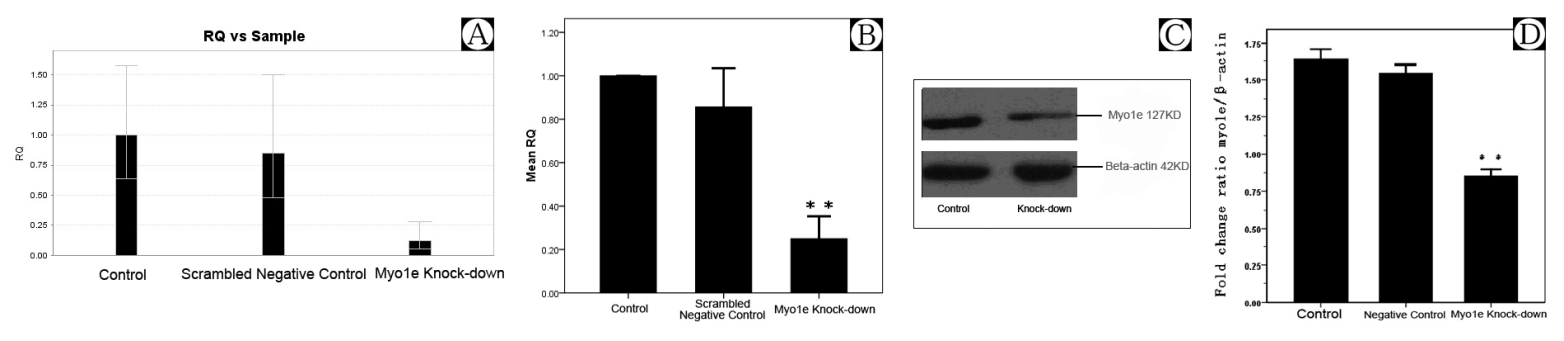
The results of *MYO1E* knock-down by real-time PCR and Western Blotting. A, B) Results of *MYO1E* knock-down by real-time PCR. C, D) The result of Western blotting of control, negative control, and *MYO1E* knock-down. **Compared with the results of control and negative control, Myo1e expression was noted to be down-regulated by real-time PCR and Western blotting, *P*<0.01.

The specifically inhibiting Myo1e in differentiated, cultured podocytes induced morphological changes with cellular elongation and loss of processes compared to the characteristic arborized phenotype (Figure 3E–G). Furthermore, these morphological changes were associated with actin cytoskeleton re-arrangement. In control podocytes, actin is distributed as parallel bundles of stress fibers and a cortical ring of filamentous actin [24]. Inhibition of Myo1e activity was associated with abolishment of these actin stress fibers while enhancement of the cortical actin web in the cytoplasm, resulting in a polygonal cellular shape (Figure 3H–J). The effects of *MYO1E* knock-down were confirmed by real-time PCR and Western blot (Figure 4).

### Down regulation of Myo1e decreased cell proliferative activity in cultured podocytes

**Figure 5:**
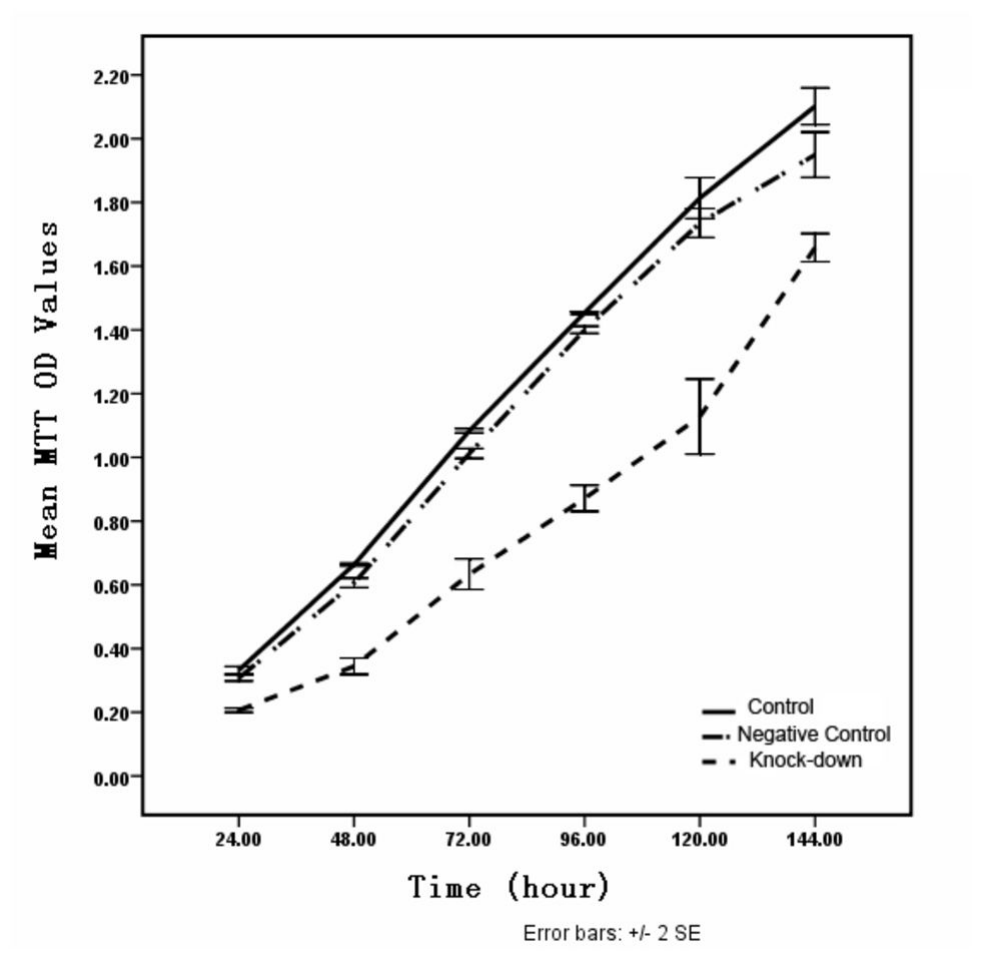
Proliferation of cultured podocytes by MTT assay. There was a significant decrease in the cell number in *MYO1E* knock-down podocytes compared to the cell number in control and scramble negative controls at 24, 48, 72, 96, 120, and 144 h. The inhibition of podocyte proliferation in the *MYO1E* knock-down group was most evident at 48 h.

Cell proliferation was measured by the MTT assay. There was a significant decrease in cell number in MYO1E knock-down podocytes compared to the results in the control and scrambled negative control groups at 24, 48, 72, 96, 120, and 144 h, respectively. The most evident inhibition of podocyte proliferation in the *MYO1E* knock-down group was seen at 48 h (Table 2 and Figure 5). Simultaneously, ELISA measurement of the results for *MYO1E* knock-down was conducted, which showed that the effect of *MYO1E* knock-down was also most evident at 48 h (data not shown). Present results implied that there could be increased cell death/loss from down-expression of Myo1e in podocytes which don’t usually proliferate.

**Table 2:** Percentage of decrease in cell number between the *MYO1E* knock-down and scrambled control groups by MTT assay.

Time point (h)	Scrambled control	*MYO1E* knock-down
24	7±1.17%	38±4.5%**
48	8±1.16%	48±2.8%**
72	6±0.58%	40±2%**
96	4±1%	40±2%**
120	4±4.5%	38±3%**
144	7±1%	25±7%**

** *P* < 0.01

### Cell migration was decreased in *MYO1E* knock-down podocytes

**Figure 6:**
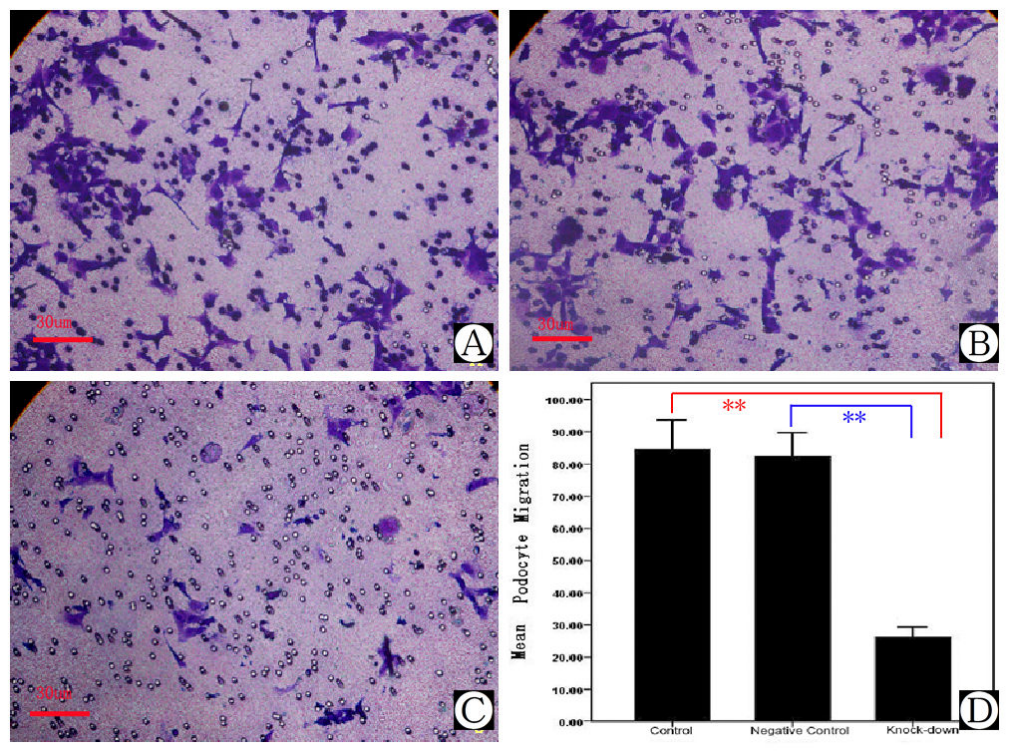
Migration assay of Myo1e depleted podocytes. A) Podocyte migration in control group. B) Podocyte migration in scrambled negative control group. C) Podocyte migration in *MYO1E* knock-down group. D) Comparison of mean migration effects of podocytes in different groups. **Compared with the results of control and negative control group, podocyte migration in the *MYO1E* knock-down group was down-regulated, *P*<0.01.

Foot processes of podocytes are highly flexible and dynamic structures, and play a key role in withstanding the continuous filtration pressure in the kidneys. In the event of damage or loss of single podocytes, the motility of the podocytes is important to repair wounds on the capillary loop. Previous studies have shown that these processes depend on rearrangements of the actin cytoskeleton [25]. Podocyte motility was measured by the transwell migration assay. Compared with the results from the control, Myo1e depletion significantly decreased podocyte motility in the *MYO1E* knock-down group (Figure 6 and Table 3, *P*<0.01, *n*=6).

**Table 3:** Transwell migration assay of *MYO1E* knock-down (mean ± SD).

Treatment	Number	RSMCs Migration
Control	6	84.67±10.94
Scrambled negative control	6	82.33±9.07
*MYO1E* knock-down	6	26.14±4.30**

** *P* < 0.001

### Myo1e depletion reduced the rate of endocytosis of FITC-transferrin in podocytes

**Figure 7:**
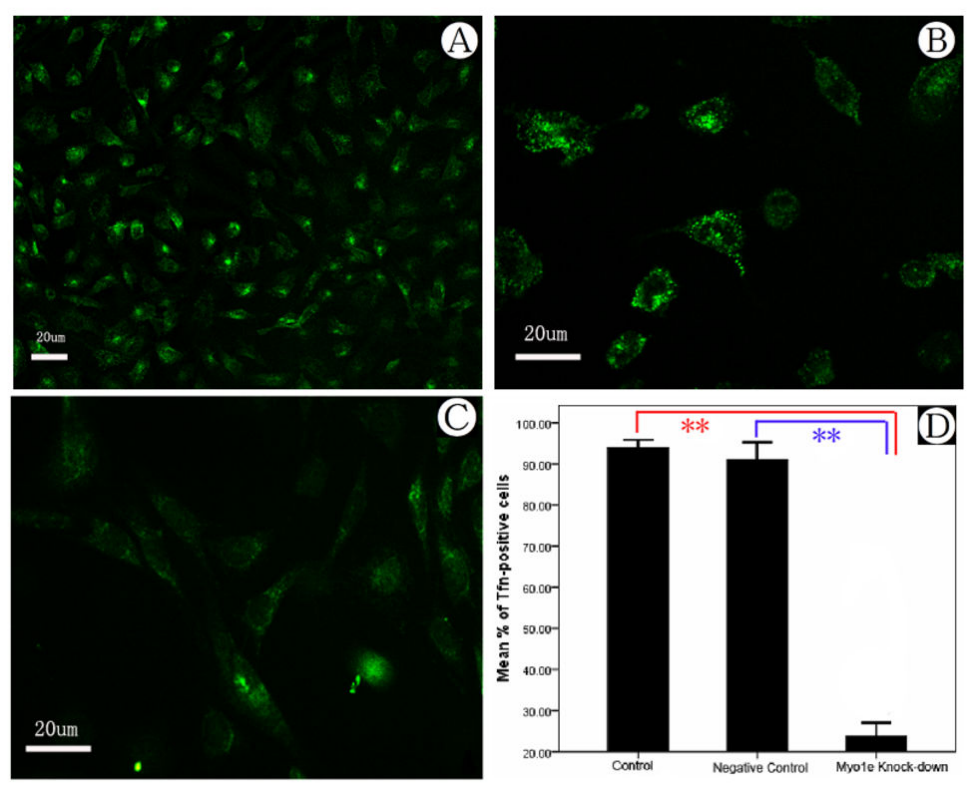
Transferrin endocytosis assay of cultured podocytes. A) Podocyte endocytosis with FITC-transferrin in control group. B) Podocyte endocytosis with FITC-transferrin in negative control group. C) Podocyte endocytosis with FITC-transferrin in *MYO1E* knock-down group. Arrow: podocytes with endocytic FITC-transferrin granules. D) Comparison of podocyte endocytosis in different groups. **Compared with the results from the control and negative control groups, podocyte endocytosis in the *MYO1E* knock-down group was down-regulated, *P*<0.01.

Mechanisms of glomerular protein handling have received much attention in the study of nephrotic syndrome, and following the recognition of proteinuria as an independent risk factor for both renal failure and cardiovascular disease. Protein endocytosis by podocytes may represent a useful, measurable phenotypic characteristic against which potentially injurious or beneficial interventions can be assessed [26]. To analyze effect of Myo1e depletion on the endocytosis function of podocyte, we used FITC-transferrin to study protein endocytosis by direct quantitative assay and fluorescence microscopy. After co-incubation with MPC5 by FITC-transferrin for 15 min, there was a dramatic decrease in the number of cells with FITC-positive vesicles within the podocyte cell bodies in the *MYO1E* knock-down group compared with control and negative control group (Figure 7).

### Cell adhesion was decreased in *MYO1E* knock-down podocytes

**Figure 8:**
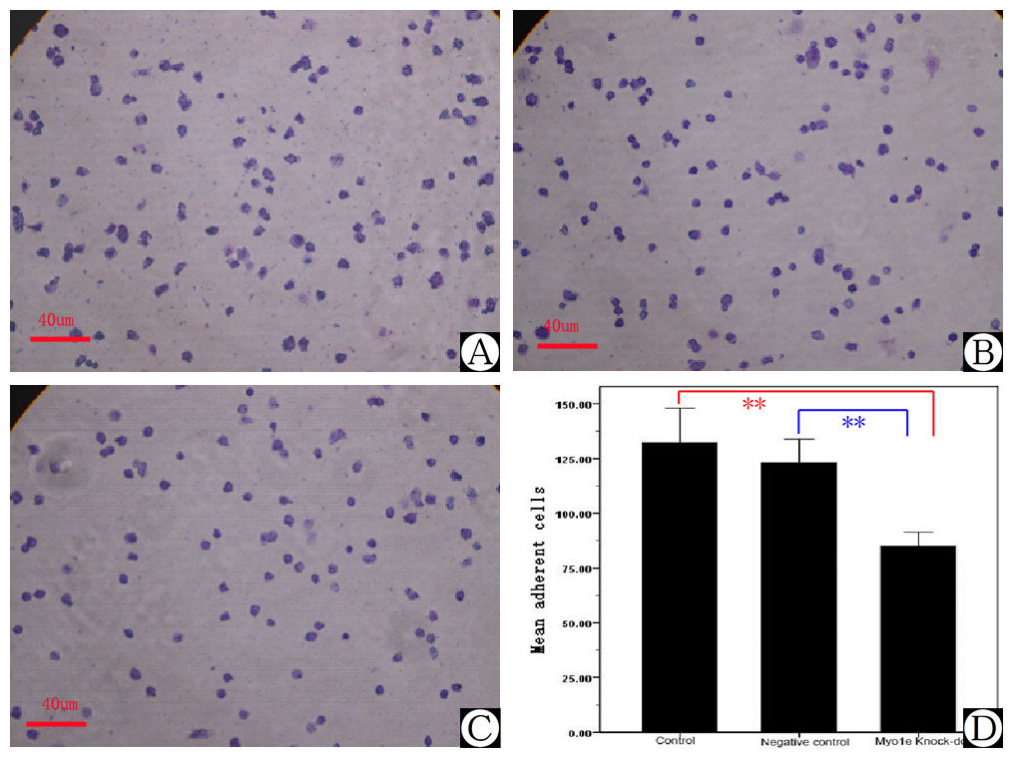
Adhesion assay of cultured podocytes. A) Podocyte adhesion in control group. B) Podocyte adhesion in the scrambled negative control group. C) Podocyte adhesion in the *MYO1E* knock-down group. D) Comparison of podocyte adhesion assay in different groups. **Compared with the results of the control and negative control groups, podocyte adhesion in the *MYO1E* knock-down group was down-regulated, *P*<0.05.

Cell-cell contacts and the adherence of podocytes to the extracellular matrix of the GBM are crucial for podocyte function. Adhesion assays were performed to investigate the effect of *MYO1E* knock-down on podocyte anchorage to the GBM. Figure 8 shows that *MYO1E* knock-down significantly reduced the adherence of podocytes (Figure 8, *P*<0.01, *n*=6).

### The down regulation of Myo1e accelerates PAN-induced podocyte detachment

**Figure 9:**
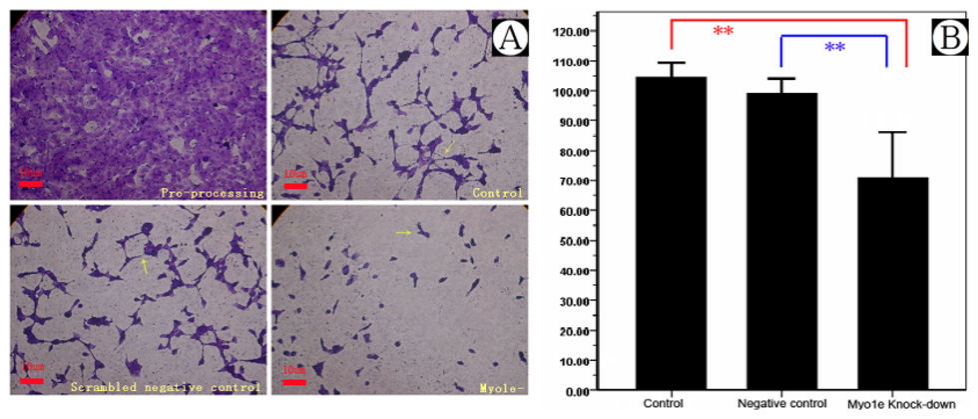
Detachment assay of cultured podocytes. A) Compared with the results before treatment, the number of cells attached on bottom of the cultural bottle was significantly decreased in the three groups after PAN treatment. This phenomenon was more evident in *MYO1E* knock-down podocytes compared with the control and negative control groups after PAN treatment. B) The differences among the control, negative control, and *MYO1E* knock-down groups. **Compared with the results in the control and negative control groups, podocyte detachment in the *MYO1E* knock-down group was down-regulated, *P*<0.01.

We compared cell detachment in control and *MYO1E* knock-down cultured podocytes. Although there was no significant difference between the PAN-treated control and *MYO1E* knock-down podocytes at 24 h after PAN treatment, we noted a significant difference in cell detachment after 48 h (*P*<0.01, *n*=6). Figure 9 shows that the cell number attached to the bottom of the culture bottle was significantly decreased in the control, scramble negative control, and *MYO1E* knock-down groups after PAN treatment compared with the results before treatment. Furthermore, this phenomenon was more evident in *MYO1E* knock-down podocytes compared with the control and negative control groups. In the control and scramble negative control groups, although the number of attached cells was reduced, the interaction of foot processes from neighbor podocytes was still observed. In the *MYO1E* knock-down group, the interactions of foot processes were replaced by foot process effacement which was normally seen in patients with nephrotic range proteinuria. Figure 9A shows the number of cells remaining on the bottom of the culture plate in the three different groups. These data suggest an attachment supporting effect of Myo1e as a key component of the cytoskeleton in cultured mouse podocytes.

## Discussion

In present study, depletion of Myo1e resulted in pericardial edema, an expanded Bowman’s space, pronephric cysts, and evidence of proteinuria in zebrafish. Furthermore, down regulation of Myo1e in cultured mouse differentiated podocytes not only resulted in morphological changes and disrupted cytoskeletal integrity with loss of actin stress fibers in the cytoplasm, but also decreased proliferation activity, reduced ability of cell migration, endocytosis, and adhesion to GBM. Ultimately, we provide evidence that Myo1e is a key component of cytoskeleton system, morphology maintenance, functional integrity of podocytes and the glomerular filtration barrier.

Actually, this is not the first study demonstrating the role of Myo1e in glomeruli and the kidney. Krendel et al. [9] reported that Myo1e localizes to podocytes in the kidney. The authors found that *MYO1E* knock-down mice exhibited proteinuria, signs of chronic renal injury, and kidney inflammation. Meanwhile, renal tissue from Myo1e-null mice demonstrated changes characteristic of glomerular disease including a thickened and disorganized glomerular basement membrane and flattened podocyte foot processes. These observations suggest that Myo1e plays an important role in podocyte function and normal glomerular filtration. Furthermore, Chase et al. [12] demonstrated that in mice with podocyte-specific knockout of *MYO1E* there were defects in the glomerular ultrastructure including foot process effacement and thickening and delamination of the glomerular basement membrane. Their results proved that loss of Myo1e in podocytes is sufficient to disrupt glomerular filtration, and to induce severe structural defects in the glomerulus.

One may then question the differences between the study of Krendel et al. [9] and the present study. Krendel et al. [9] demonstrated that *MYO1E* knock-down mice developed defects in glomerular organization, glomerular filtration defects, and kidney disease, while in present study we revealed that *MYO1E* knock-down zebrafish showed pericardial edema, pronephric cysts, an expanded Bowman’s space, and evidence of proteinuria. More importantly, Chase et al. [12] implied that proteinuria, the loss of glomerular integrity and glomerulosclerosis in mice with specific knockout of *MYO1E* in podocytes may be the result of subtle changes in the regulation of podocyte adhesion and cytoskeletal organization. In present study we provided direct evidence that knock-down of *MYO1E* in mouse podocytes induces actin cytoskeleton reorganization, dysfunction in cellular membrane trafficking, and adhesion to the basement membrane. Our results suggest that Myo1e dysfunction might lead to shape alteration and functional impairment in podocytes, impairment of the glomerular filtration barrier, and finally proteinuria and kidney disease via actin cytoskeleton rearrangement.

Interestingly, Mele et al. [11] recently identified two homozygous mutations (A159P and Y695X in *MYO1E*) in patients with steroid resistant nephrotic syndrome and focal segmental glomerulosclerosis from two independent pedigrees. At same time, by homozygosity mapping and exome sequencing in a consanguineous pedigree with three affected siblings, Sanna-Cherchi et al. [10] detected an A159P substitution which disrupts highly conserved protein sequences and impairs ligand binding and actin interaction in the Myo1e motor domain. The two pedigrees from each index family in both studies are so similar that we are tempted to guess that the DNA samples used may be from the same family. Even so, these studies provide strong clinical evidence of the important role of Myo1e in the maintenance of podocyte functional integrity and glomerular filtration barrier. The behavior of the abnormal Myo1e protein also provides a hint as to why agents that stabilize the podocyte cytoskeleton (e.g., cyclosporine) are important to the successful therapy of focal segmental glomerulosclerosis [27].

As a membrane-bound protein in the cytoplasm, and co-localized with CD2-associated protein (foot process molecule also involved in cytoskeletal remodeling) [27], Myo1e is speculated to play a key role in maintaining foot process architecture and slit diaphragm organization [12]. Moreover, Myo1e may be necessary for interactions between podocytes and the GBM. As we know, podocyte foot processes (FPs) are connected to the GBM [28] by two different sets of cell matrix adhesion complexes: integrins and dystroglycans [29,30]. α3β1-Integrin, localized exclusively to the podocyte’s basal membrane domains, links the GBM to podocytes by an intracellular actin cytoskeleton through a set of integrin- and actin-associated proteins that include paxillin, talin, vinculin, α-actinin-4, and filamin. Meanwhile, α- and β-dystroglycan, ubiquitously expressed in many cell types such as skeletal muscle and antigen-presenting dendritic cells, localize to the basal membrane domain in human podocytes and link the extracellular matrix to the cell cytoskeleton of podocytes [30].

In present study, lack of Myo1e impaired the cell-matrix adhesion of podocytes because Myo1e depletion resulted in decreased adhesion and aggregated puromycin aminonucleoside (PAN)-induced podocyte detachment, which leads to podocyte depletion or drop out from the GBM, and finally reduce podocyte density, resulting in an impaired glomerular filtration barrier and development of proteinuria [31]. Myo1e seems to primarily serve as an adhesion adaptor linking the actin skeleton to the GBM, or has the role of strengthening GBM adhesion in podocytes. Though the exact mechanism is not known, integrins [32] or integrin-linked kinase (ILK) [29] may be candidate molecules for further study.

## Conclusions

In summary, Myo1e depletion resulted in pericardial edema, an expanded Bowman’s space, pronephric cysts, and proteinuria in zebrafish, while it caused morphological changes, disrupted cytoskeletal integrity, and decreased the ability of proliferation, migration, endocytosis, and adhesion to the GBM in cultured mouse podocyte. These results demonstrate that Myo1e plays a key role in the physiological integrity of podocytes and the glomerular filtration barrier.

## Supporting Information

Figure S1 **Myo1e expression in Heart (A), Liver (B) and Spleen (C) tissues (scale bar 100μm).** D. For negative control by adding secondary antibody only in Myo1e staining in glomerular samples.
(JPG)
