# Supplementary figures and images for: Myo1e Impairment Results in Actin Reorganization, Podocyte Dysfunction, and Proteinuria in Zebrafish and Cultured Podocytes
Source: PLoS One. 2013 Aug 19;8(8):e72750. doi: 10.1371/journal.pone.0072750 (PMC3747079; doi:10.1371/journal.pone.0072750)

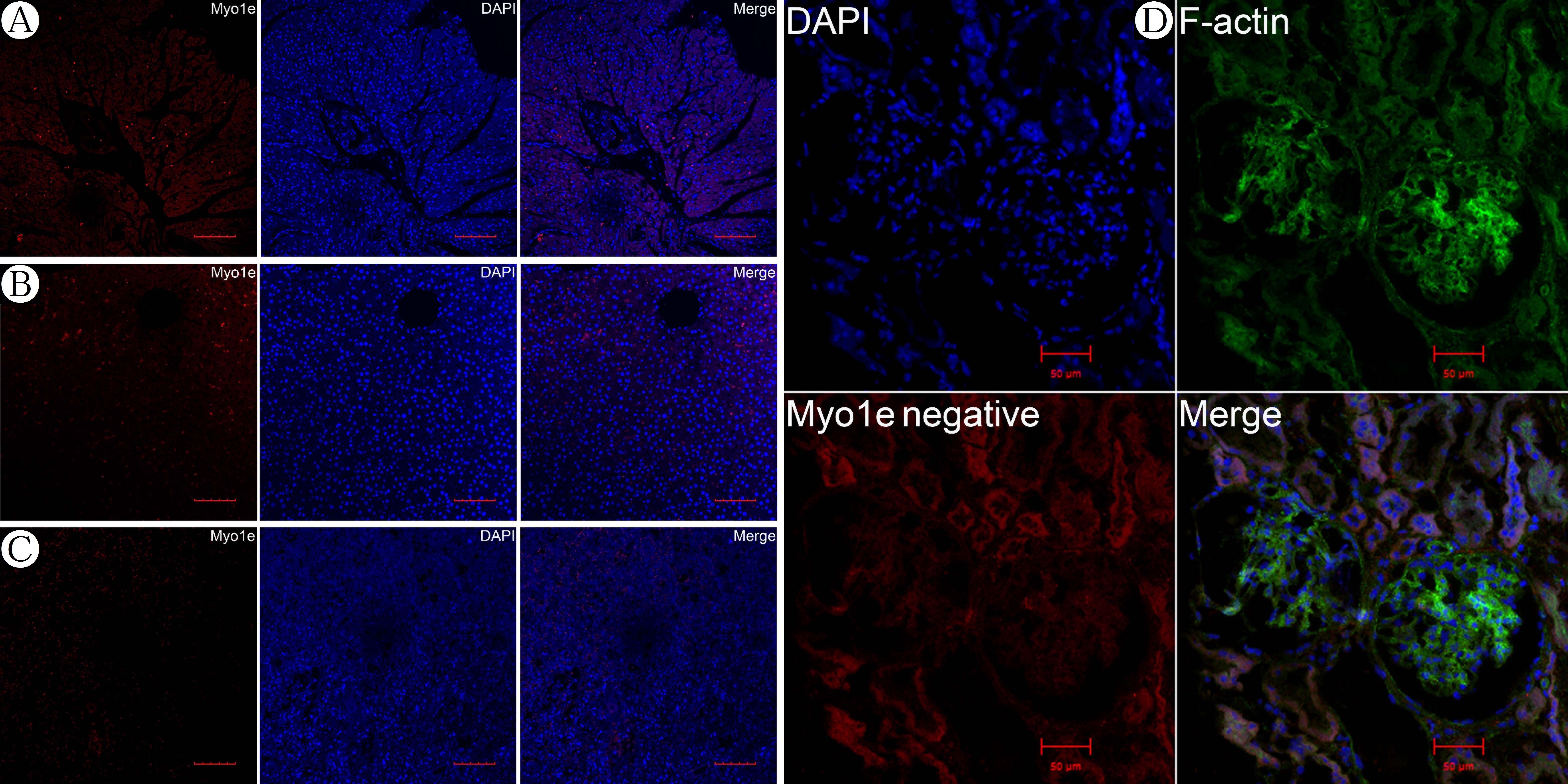

Supplement: Figure S1 — Myo1e expression in Heart (A), Liver (B) and Spleen (C) tissues (scale bar 100μm). D. For negative control by adding secondary antibody only in Myo1e staining in glomerular samples. (JPG) [file pone.0072750.s001.jpg]
